# Bis(2-hy­droxy­ethanaminium) biphenyl-4,4′-dicarboxyl­ate

**DOI:** 10.1107/S1600536811013523

**Published:** 2011-05-07

**Authors:** Bin Deng, Rui-Jin Yu

**Affiliations:** a Department of Chemistry and Life Sciences, Xiangnan University, Chenzhou 423000, People’s Republic of China; b College of Science, Northwest A&F University, Yangling, Shaanxi 712100, People’s Republic of China

## Abstract

In the title compound, 2C_2_H_8_NO^+^·C_14_H_8_O_4_^2−^, the dihedral angle between the benzene rings of the dianion is 9.95 (12)°. In the crystal, the cations and anions are linked *via* inter­molecular O—H⋯O and N—H⋯O hydrogen bonds, generating layers lying parallel to (001).

## Related literature

For backround to organic salts, see: Holman *et al.* (2001 ▶); Plaut *et al.* (2000 ▶); Russell *et al.* (1997 ▶). For hydrogen-bond networks in related compounds, see; Ranganathan *et al.*, (1999 ▶); Swift *et al.* (1998 ▶); Zhang & Chen (2005 ▶); Bhogala & Nangia (2003 ▶). For hydrogen-bond graph-set motifs, see: Bernstein *et al.* (1995 ▶).
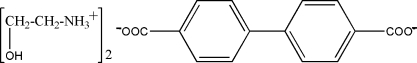


## Experimental

### 

#### Crystal data

2C_2_H_8_NO^+^·C_14_H_8_O_4_^2−^
*M* *_r_* = 364.39
Orthorhombic, 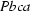

*a* = 7.3410 (7) Å
*b* = 12.4094 (13) Å
*c* = 38.074 (4) Å
*V* = 3468.5 (6) Å^3^
*Z* = 8
Mo *K*α radiation
μ = 0.11 mm^−1^
*T* = 293 K
0.30 × 0.27 × 0.18 mm

#### Data collection

Bruker SMART CCD diffractometer
Absorption correction: multi-scan (*SADABS*; Sheldrick, 1996 ▶) *T*_min_ = 0.969, *T*_max_ = 0.981
14999 measured reflections
3380 independent reflections
2606 reflections with *I* > 2σ(*I*)
*R*_int_ = 0.044

#### Refinement

*R*[*F* ^2^ > 2σ(*F* ^2^)] = 0.066
*wR*(*F* ^2^) = 0.173
*S* = 1.08
3380 reflections
267 parameters
H atoms treated by a mixture of independent and constrained refinement
Δρ_max_ = 0.47 e Å^−3^
Δρ_min_ = −0.18 e Å^−3^

### 

Data collection: *SMART* (Bruker, 1998 ▶); cell refinement: *SAINT* (Bruker, 1999 ▶); data reduction: *SAINT*; program(s) used to solve structure: *SHELXS97* (Sheldrick, 2008 ▶); program(s) used to refine structure: *SHELXL97* (Sheldrick, 2008 ▶); molecular graphics: *SHELXTL* (Sheldrick, 2008 ▶); software used to prepare material for publication: *SHELXTL*.

## Supplementary Material

Crystal structure: contains datablocks I, global. DOI: 10.1107/S1600536811013523/lh5226sup1.cif

Structure factors: contains datablocks I. DOI: 10.1107/S1600536811013523/lh5226Isup2.hkl

Additional supplementary materials:  crystallographic information; 3D view; checkCIF report

## Figures and Tables

**Table 1:** Hydrogen-bond geometry (Å, °)

*D*—H⋯*A*	*D*—H	H⋯*A*	*D*⋯*A*	*D*—H⋯*A*
O5—H5*O*⋯O1	0.87 (4)	1.94 (4)	2.793 (3)	165 (4)
N1—H1*A*⋯O2	0.96 (4)	1.76 (4)	2.704 (3)	169 (3)
N1—H1*B*⋯O3^i^	0.94 (4)	1.87 (4)	2.807 (3)	180 (4)
N1—H1*C*⋯O1^ii^	0.90 (4)	2.09 (4)	2.892 (3)	149 (3)
O6—H6*O*⋯O4	0.92 (4)	1.89 (4)	2.778 (3)	164 (4)
N2—H2*A*⋯O3	0.95 (3)	1.82 (4)	2.759 (3)	170 (3)
N2—H2*B*⋯O4^iii^	0.95 (4)	2.00 (4)	2.854 (3)	149 (3)
N2—H2*C*⋯O1^iv^	0.95 (4)	1.91 (4)	2.866 (3)	174 (4)

Symmetry codes: (i) 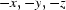; (ii) 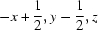; (iii) 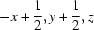; (iv) 
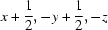
.

**Fig. 1.**
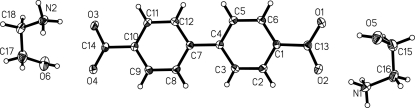
The asymmetric unit of the title compound with displacement ellipsoids drawn at the 30% probability level.

**Fig. 2.**
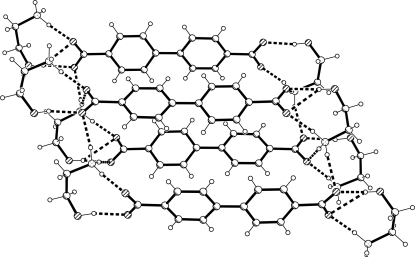
Part of the crystal structure showing a two-dimensional hydrogen-bonded (dashed lines) layer parallel to (001).

## supplementary crystallographic information

### Comment

Organic crystals built from acid-base complexes have received much attention in the predictable assembly of supramolecular architectures (Holman *et al.*, 2001; Plaut *et al.*, 2000; Russell *et al.*, 1997). One of the most important applications, is the use of self-assembly of small molecules with O—H···O, N—H···O and other weaker intermolecular interactions to create one-, two- and three-dimensional networks (Ranganathan *et al.*, 1999; Swift *et al.*, 1998; Zhang *et al.*, 2005). Aromatic acids have attracted our interest because of their importance in crystal engineering and they can form strong directional hydrogen bonds (Bhogala & Nangia, 2003). It is known that ethanolamine (ED) is a good organic base and has hydrogen-bond donor sites. Therefore, combinations of 4,4'-dicarboxyl-biphenyl(BDB) with ethanolamine molecules may be expected to display an interesting network. Herein, we report the crystal structure of the title organic salt (I).

The asymmetric unit of (I) is composed of two independent ED+ cations and one BDB^2-^ dianion (Fig. 1). The protons of two carboxyl groups from BDB are transferred to the amido groups of ED. The dihedral angle between the two benzene rings of the BDB^2-^ dianion is  9.95 (12) °. In the crystal, dianions and cations are linked via intermolecular O—H···O and N—H···O hydrogen bonds to form a two-dimensional network parallel to (001) which includes R^2^_2_(9) rings (Bernstein *et al.*, 1995).

### Experimental

BDB (0.024 g, 0.01 mmol) and ED (0.012 g, 0.02 mol) were dissolved in hot EtOH/H_2_O (1:1) solution (20 mL). The solution was allowed to cool to room temperature and was evaporated in air for 4 days to give colorless crystals of the title compound (yield: 23%).

### Refinement

H atoms bonded to C atoms were positioned geometrically and refined as riding atoms, with C—H = 0.93 - 0.97Å and *U*_iso_(H) = 1.2*U*_eq_(C). H atoms bonded to N and O atoms were refined independently with isotropic displacement parameters.

### Crystal data

**Table:**

2C_2_H_8_NO^+^·C_14_H_8_O_4_^2^^−^	*F*(000) = 1552
*M**_r_* = 364.39	*D*_x_ = 1.396 Mg m^−^^3^
Orthorhombic, *P**b**c**a*	Mo *K*α radiation, λ = 0.71073 Å
Hall symbol: -P 2ac 2ab	Cell parameters from 3380 reflections
*a* = 7.3410 (7) Å	θ = 2.1–26.0°
*b* = 12.4094 (13) Å	µ = 0.11 mm^−^^1^
*c* = 38.074 (4) Å	*T* = 293 K
*V* = 3468.5 (6) Å^3^	Block, colorless
*Z* = 8	0.30 × 0.27 × 0.18 mm

### Data collection

**Table:**

Bruker SMART CCD diffractometer	3380 independent reflections
Radiation source: fine-focus sealed tube	2606 reflections with *I* > 2σ(*I*)
graphite	*R*_int_ = 0.044
φ and ω scans	θ_max_ = 26.0°, θ_min_ = 2.1°
Absorption correction: multi-scan (*SADABS*; Sheldrick, 1996)	*h* = −7→9
*T*_min_ = 0.969, *T*_max_ = 0.981	*k* = −15→14
14999 measured reflections	*l* = −27→46

### Refinement

**Table:**

Refinement on *F*^2^	Primary atom site location: structure-invariant direct methods
Least-squares matrix: full	Secondary atom site location: difference Fourier map
*R*[*F*^2^ > 2σ(*F*^2^)] = 0.066	Hydrogen site location: inferred from neighbouring sites
*wR*(*F*^2^) = 0.173	H atoms treated by a mixture of independent and constrained refinement
*S* = 1.08	*w* = 1/[σ^2^(*F*_o_^2^) + (0.0845*P*)^2^ + 1.5997*P*] where *P* = (*F*_o_^2^ + 2*F*_c_^2^)/3
3380 reflections	(Δ/σ)_max_ < 0.001
267 parameters	Δρ_max_ = 0.47 e Å^−^^3^
0 restraints	Δρ_min_ = −0.18 e Å^−^^3^

### Special details

**Table:**

Geometry. All e.s.d.'s (except the e.s.d. in the dihedral angle between two l.s. planes) are estimated using the full covariance matrix. The cell e.s.d.'s are taken into account individually in the estimation of e.s.d.'s in distances, angles and torsion angles; correlations between e.s.d.'s in cell parameters are only used when they are defined by crystal symmetry. An approximate (isotropic) treatment of cell e.s.d.'s is used for estimating e.s.d.'s involving l.s. planes.
Refinement. Refinement of *F*^2^ against ALL reflections. The weighted *R*-factor *wR* and goodness of fit *S* are based on *F*^2^, conventional *R*-factors *R* are based on *F*, with *F* set to zero for negative *F*^2^. The threshold expression of *F*^2^ > σ(*F*^2^) is used only for calculating *R*-factors(gt) *etc*. and is not relevant to the choice of reflections for refinement. *R*-factors based on *F*^2^ are statistically about twice as large as those based on *F*, and *R*- factors based on ALL data will be even larger.

### Fractional atomic coordinates and isotropic or equivalent isotropic displacement parameters (Å^2^)

**Table:**

	*x*	*y*	*z*	*U*_iso_*/*U*_eq_	
O1	0.0874 (3)	−0.00499 (14)	−0.14379 (4)	0.0385 (5)	
O2	0.3194 (3)	−0.10392 (16)	−0.12492 (5)	0.0489 (6)	
O3	0.0498 (3)	0.35559 (14)	0.13128 (5)	0.0377 (5)	
O4	0.2920 (3)	0.25707 (14)	0.14555 (4)	0.0384 (5)	
C1	0.1729 (3)	0.00670 (18)	−0.08337 (6)	0.0264 (5)	
C2	0.2609 (4)	−0.0429 (2)	−0.05568 (6)	0.0319 (6)	
H2	0.3272	−0.1056	−0.0597	0.038*	
C3	0.2524 (3)	−0.0012 (2)	−0.02219 (6)	0.0310 (6)	
H3	0.3124	−0.0365	−0.0040	0.037*	
C4	0.1558 (3)	0.09265 (18)	−0.01495 (6)	0.0240 (5)	
C5	0.0640 (4)	0.1404 (2)	−0.04283 (6)	0.0337 (6)	
H5	−0.0037	0.2026	−0.0389	0.040*	
C6	0.0711 (4)	0.0977 (2)	−0.07628 (6)	0.0339 (6)	
H6	0.0063	0.1307	−0.0943	0.041*	
C7	0.1556 (3)	0.14109 (18)	0.02092 (6)	0.0256 (5)	
C8	0.2715 (4)	0.1027 (2)	0.04696 (6)	0.0329 (6)	
H8	0.3465	0.0441	0.0421	0.039*	
C9	0.2776 (4)	0.1497 (2)	0.07989 (6)	0.0324 (6)	
H9	0.3594	0.1239	0.0965	0.039*	
C10	0.1640 (3)	0.23463 (18)	0.08846 (6)	0.0263 (5)	
C11	0.0479 (4)	0.2731 (2)	0.06293 (7)	0.0362 (6)	
H11	−0.0299	0.3300	0.0681	0.043*	
C12	0.0455 (4)	0.2282 (2)	0.02960 (7)	0.0374 (7)	
H12	−0.0316	0.2571	0.0127	0.045*	
C13	0.1935 (4)	−0.03813 (19)	−0.11985 (6)	0.0311 (6)	
C14	0.1693 (3)	0.28548 (19)	0.12432 (6)	0.0290 (6)	
O5	0.2304 (3)	−0.09198 (17)	−0.20556 (6)	0.0521 (6)	
H5O	0.206 (5)	−0.066 (3)	−0.1849 (10)	0.081 (13)*	
C15	0.1517 (5)	−0.1945 (2)	−0.21076 (8)	0.0503 (8)	
H15A	0.1241	−0.2034	−0.2355	0.060*	
H15B	0.0378	−0.1982	−0.1979	0.060*	
C16	0.2713 (5)	−0.2841 (2)	−0.19924 (7)	0.0480 (8)	
H16A	0.2242	−0.3515	−0.2084	0.058*	
H16B	0.3923	−0.2735	−0.2089	0.058*	
N1	0.2835 (4)	−0.2910 (2)	−0.16066 (6)	0.0386 (6)	
H1A	0.310 (4)	−0.224 (3)	−0.1494 (9)	0.059 (10)*	
H1B	0.172 (5)	−0.313 (3)	−0.1509 (9)	0.056 (10)*	
H1C	0.362 (5)	−0.342 (3)	−0.1539 (9)	0.059 (10)*	
O6	0.2281 (4)	0.34359 (17)	0.21172 (6)	0.0619 (7)	
H6O	0.267 (6)	0.324 (3)	0.1898 (11)	0.092 (14)*	
C17	0.3001 (5)	0.4453 (2)	0.21715 (8)	0.0549 (9)	
H17A	0.4237	0.4461	0.2082	0.066*	
H17B	0.3067	0.4580	0.2423	0.066*	
C18	0.1995 (4)	0.5355 (2)	0.20099 (7)	0.0406 (7)	
H18A	0.2489	0.6032	0.2095	0.049*	
H18B	0.0726	0.5319	0.2080	0.049*	
N2	0.2115 (4)	0.5327 (2)	0.16204 (6)	0.0379 (6)	
H2A	0.145 (4)	0.473 (3)	0.1534 (8)	0.059 (9)*	
H2B	0.168 (5)	0.600 (3)	0.1536 (9)	0.070 (11)*	
H2C	0.336 (6)	0.527 (3)	0.1547 (10)	0.084 (13)*	

### Atomic displacement parameters (Å^2^)

**Table:**

	*U*^11^	*U*^22^	*U*^33^	*U*^12^	*U*^13^	*U*^23^
O1	0.0438 (11)	0.0429 (11)	0.0287 (10)	0.0014 (9)	−0.0091 (9)	−0.0037 (7)
O2	0.0548 (13)	0.0513 (12)	0.0405 (11)	0.0195 (11)	−0.0082 (10)	−0.0194 (9)
O3	0.0385 (11)	0.0364 (10)	0.0383 (10)	0.0059 (9)	0.0008 (8)	−0.0135 (8)
O4	0.0477 (12)	0.0380 (10)	0.0294 (9)	0.0072 (9)	−0.0068 (8)	−0.0062 (7)
C1	0.0265 (13)	0.0271 (12)	0.0257 (12)	−0.0037 (10)	−0.0016 (10)	−0.0020 (9)
C2	0.0346 (14)	0.0258 (12)	0.0353 (14)	0.0048 (11)	−0.0031 (11)	−0.0032 (10)
C3	0.0348 (14)	0.0314 (13)	0.0267 (12)	0.0051 (11)	−0.0069 (11)	0.0004 (10)
C4	0.0216 (12)	0.0245 (12)	0.0258 (12)	−0.0041 (10)	0.0017 (9)	−0.0005 (9)
C5	0.0357 (15)	0.0326 (13)	0.0329 (14)	0.0107 (12)	−0.0011 (11)	−0.0033 (10)
C6	0.0370 (15)	0.0385 (14)	0.0262 (13)	0.0105 (12)	−0.0057 (11)	0.0009 (10)
C7	0.0240 (13)	0.0253 (12)	0.0277 (13)	−0.0019 (10)	0.0008 (10)	−0.0018 (9)
C8	0.0359 (15)	0.0332 (14)	0.0295 (13)	0.0116 (12)	0.0010 (11)	−0.0027 (10)
C9	0.0363 (15)	0.0376 (14)	0.0234 (12)	0.0064 (12)	−0.0019 (11)	0.0001 (10)
C10	0.0268 (13)	0.0259 (12)	0.0263 (12)	−0.0035 (10)	0.0032 (10)	−0.0023 (9)
C11	0.0393 (16)	0.0327 (14)	0.0367 (14)	0.0104 (12)	−0.0026 (12)	−0.0099 (11)
C12	0.0406 (16)	0.0403 (15)	0.0312 (14)	0.0124 (13)	−0.0116 (12)	−0.0052 (11)
C13	0.0352 (15)	0.0264 (12)	0.0317 (13)	−0.0035 (11)	−0.0037 (11)	−0.0039 (10)
C14	0.0327 (14)	0.0250 (12)	0.0293 (13)	−0.0057 (11)	0.0042 (11)	−0.0024 (10)
O5	0.0747 (16)	0.0416 (12)	0.0399 (12)	−0.0011 (11)	0.0125 (11)	0.0027 (9)
C15	0.062 (2)	0.0510 (19)	0.0379 (16)	0.0007 (16)	−0.0091 (15)	−0.0050 (13)
C16	0.062 (2)	0.0407 (16)	0.0413 (16)	0.0055 (15)	0.0064 (15)	−0.0114 (12)
N1	0.0397 (15)	0.0317 (13)	0.0445 (14)	0.0062 (12)	0.0061 (12)	−0.0016 (11)
O6	0.108 (2)	0.0403 (12)	0.0376 (12)	−0.0153 (13)	0.0034 (13)	0.0029 (9)
C17	0.082 (3)	0.0512 (19)	0.0319 (15)	−0.0059 (18)	−0.0003 (16)	−0.0017 (13)
C18	0.0464 (18)	0.0347 (15)	0.0407 (15)	−0.0025 (13)	0.0095 (13)	−0.0067 (11)
N2	0.0474 (16)	0.0274 (12)	0.0389 (13)	0.0002 (11)	0.0018 (12)	0.0014 (10)

### Geometric parameters (Å, °)

**Table:**

O1—C13	1.268 (3)	C11—H11	0.9300
O2—C13	1.248 (3)	C12—H12	0.9300
O3—C14	1.264 (3)	O5—C15	1.411 (4)
O4—C14	1.261 (3)	O5—H5O	0.87 (4)
C1—C6	1.381 (3)	C15—C16	1.483 (4)
C1—C2	1.381 (3)	C15—H15A	0.9700
C1—C13	1.504 (3)	C15—H15B	0.9700
C2—C3	1.378 (3)	C16—N1	1.474 (4)
C2—H2	0.9300	C16—H16A	0.9700
C3—C4	1.391 (3)	C16—H16B	0.9700
C3—H3	0.9300	N1—H1A	0.96 (4)
C4—C5	1.390 (3)	N1—H1B	0.94 (4)
C4—C7	1.492 (3)	N1—H1C	0.90 (4)
C5—C6	1.381 (3)	O6—C17	1.384 (4)
C5—H5	0.9300	O6—H6O	0.92 (4)
C6—H6	0.9300	C17—C18	1.475 (4)
C7—C12	1.390 (3)	C17—H17A	0.9700
C7—C8	1.391 (3)	C17—H17B	0.9700
C8—C9	1.384 (3)	C18—N2	1.486 (4)
C8—H8	0.9300	C18—H18A	0.9700
C9—C10	1.383 (3)	C18—H18B	0.9700
C9—H9	0.9300	N2—H2A	0.95 (3)
C10—C11	1.378 (3)	N2—H2B	0.95 (4)
C10—C14	1.505 (3)	N2—H2C	0.95 (4)
C11—C12	1.386 (3)		
			
C6—C1—C2	117.9 (2)	O4—C14—C10	118.9 (2)
C6—C1—C13	122.5 (2)	O3—C14—C10	117.5 (2)
C2—C1—C13	119.5 (2)	C15—O5—H5O	112 (3)
C3—C2—C1	121.2 (2)	O5—C15—C16	113.1 (3)
C3—C2—H2	119.4	O5—C15—H15A	109.0
C1—C2—H2	119.4	C16—C15—H15A	109.0
C2—C3—C4	121.4 (2)	O5—C15—H15B	109.0
C2—C3—H3	119.3	C16—C15—H15B	109.0
C4—C3—H3	119.3	H15A—C15—H15B	107.8
C5—C4—C3	116.9 (2)	N1—C16—C15	112.0 (2)
C5—C4—C7	121.8 (2)	N1—C16—H16A	109.2
C3—C4—C7	121.3 (2)	C15—C16—H16A	109.2
C6—C5—C4	121.5 (2)	N1—C16—H16B	109.2
C6—C5—H5	119.3	C15—C16—H16B	109.2
C4—C5—H5	119.3	H16A—C16—H16B	107.9
C1—C6—C5	121.0 (2)	C16—N1—H1A	114 (2)
C1—C6—H6	119.5	C16—N1—H1B	111 (2)
C5—C6—H6	119.5	H1A—N1—H1B	105 (3)
C12—C7—C8	116.9 (2)	C16—N1—H1C	111 (2)
C12—C7—C4	122.1 (2)	H1A—N1—H1C	111 (3)
C8—C7—C4	120.9 (2)	H1B—N1—H1C	104 (3)
C9—C8—C7	121.4 (2)	C17—O6—H6O	105 (3)
C9—C8—H8	119.3	O6—C17—C18	116.0 (3)
C7—C8—H8	119.3	O6—C17—H17A	108.3
C10—C9—C8	121.0 (2)	C18—C17—H17A	108.3
C10—C9—H9	119.5	O6—C17—H17B	108.3
C8—C9—H9	119.5	C18—C17—H17B	108.3
C11—C10—C9	118.1 (2)	H17A—C17—H17B	107.4
C11—C10—C14	120.7 (2)	C17—C18—N2	111.6 (2)
C9—C10—C14	121.2 (2)	C17—C18—H18A	109.3
C10—C11—C12	121.0 (2)	N2—C18—H18A	109.3
C10—C11—H11	119.5	C17—C18—H18B	109.3
C12—C11—H11	119.5	N2—C18—H18B	109.3
C11—C12—C7	121.5 (2)	H18A—C18—H18B	108.0
C11—C12—H12	119.2	C18—N2—H2A	109 (2)
C7—C12—H12	119.2	C18—N2—H2B	107 (2)
O2—C13—O1	123.8 (2)	H2A—N2—H2B	113 (3)
O2—C13—C1	117.3 (2)	C18—N2—H2C	110 (2)
O1—C13—C1	118.8 (2)	H2A—N2—H2C	109 (3)
O4—C14—O3	123.6 (2)	H2B—N2—H2C	107 (3)

### Hydrogen-bond geometry (Å, °)

**Table:**

*D*—H···*A*	*D*—H	H···*A*	*D*···*A*	*D*—H···*A*
O5—H5O···O1	0.87 (4)	1.94 (4)	2.793 (3)	165 (4)
N1—H1A···O2	0.96 (4)	1.76 (4)	2.704 (3)	169 (3)
N1—H1B···O3^i^	0.94 (4)	1.87 (4)	2.807 (3)	180 (4)
N1—H1C···O1^ii^	0.90 (4)	2.09 (4)	2.892 (3)	149 (3)
O6—H6O···O4	0.92 (4)	1.89 (4)	2.778 (3)	164 (4)
N2—H2A···O3	0.95 (3)	1.82 (4)	2.759 (3)	170 (3)
N2—H2B···O4^iii^	0.95 (4)	2.00 (4)	2.854 (3)	149 (3)
N2—H2C···O1^iv^	0.95 (4)	1.91 (4)	2.866 (3)	174 (4)

Symmetry codes: (i) −*x*, −*y*, −*z*; (ii) −*x*+1/2, *y*−1/2, *z*; (iii) −*x*+1/2, *y*+1/2, *z*; (iv) *x*+1/2, −*y*+1/2, −*z*.
